# Failing to fail phenomena

**DOI:** 10.1111/eje.12768

**Published:** 2022-01-22

**Authors:** Carly Dixon, Reza Vahid Roudsari

**Affiliations:** ^1^ The University of Manchester Division of Dentistry Clinical Lecturer in Paediatric Dentistry Manchester UK; ^2^ The University of Manchester Division of Dentistry Professor and Hon Consultant in Restorative Dentistry Manchester UK

**Keywords:** competence‐based assessment, dental education, dental training, longitudinal assessment

## Abstract

**Introduction:**

Clinical competence is the backbone of competence‐based dental education. Over time, there has been a paradigm shift toward training students who are capable of independent practice, as opposed to mere academic success.

**Methods:**

A mixed‐method study was undertaken by anonymised email questionnaire to all restorative tutors at the UK Dental School. Demographics and teaching experience were ascertained, along with key questions on the utilisation of online assessment software iDentity. The assessment process for tutors was explored, and barriers experienced when grading students were reported.

**Results:**

The questionnaire was sent to all 51 restorative tutors with a response rate of 59% (*n* = 30). Only 3.5% of tutors provided verbal feedback and grading to students in person, with 20.7% only completing iDentity gradings following an email reminder. The majority of staff (93.3%) felt comfortable in raising concerns; however, one of the three clinical tutors admitted they had allowed a failing student to a pass. Qualitative analysis demonstrated several themes why tutors were reluctant to fail students: maintaining good relationships, limited supervision, time delay of grading, one‐off event and the student's first attempt.

**Conclusions:**

Grading students as competent as a one‐off experience could potentially mask a recurring problem with a student, in turn impacting the student's ability to assess their own weakness and believe themselves to be competent, and potentially be overconfident. Fair and accurate assessment has a significant benefit to student and staff, enabling targeted development to motivate the students and improve the quality of care provided to the patients.

## 1 INTRODUCTION

Achieving clinical competence is the backbone of competence‐based education (CBE). Over the past decade, regulators of clinical degrees have been on a paradigm shift toward enforcing production of graduates who are capable of independent clinical practice as opposed to individuals who are merely academically successful.^1^, ^2^, ^3^, ^4^, ^5^, ^6^, ^7^, ^8^, ^9^ This shift has been fuelled by the change in perception whereby “expertise” surpasses “experience”^10^, ^11^ and is aligned to societal and patient needs.^12^

**FIGURE 1:**
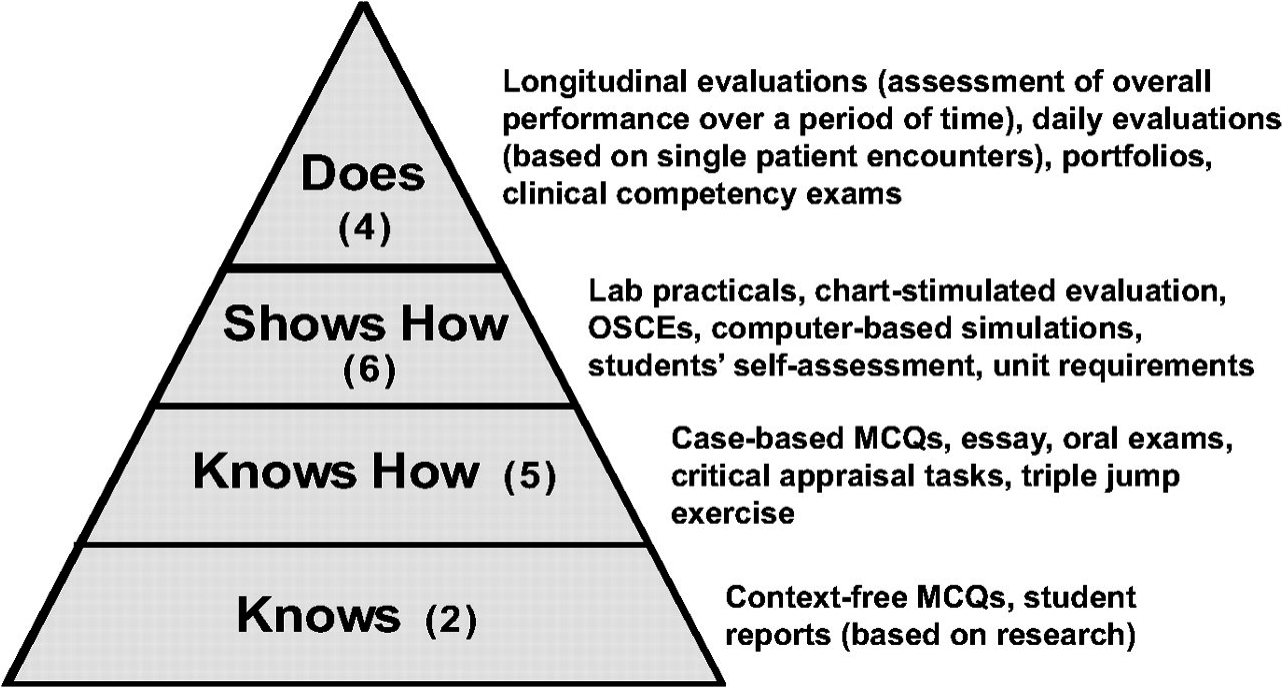
Distribution of seventeen assessment methods in the 2008 survey of assessment practices in US dental schools amongst Miller's pyramid of professional competence levels

Miller.^13^ proposed four stages for achieving clinical competence, placing “competence” at the peak of its pyramid (Figure 1). A student at the ‘does’ level not only has acquired all necessary knowledge in a given field and has been able to make pragmatic connections between such pieces of information, but also is capable of demonstrating how a procedure is done and is able to perform such procedures on their own.

At the heart of CBE lies the assessment. It is crucial for the educators to be able to demonstrate how and when a student progresses from one level of the pyramid to another. Consequently, the literature has been exponentially expanding on this topic.^14^, ^15^, ^16^, ^17^, ^18^, ^19^, ^20^, ^21^ Out of a variety of toolkits available in the toolbox of assessment, longitudinal assessment of performance over time has been described as an optimal method at the highest level of clinical competence.^22^, ^23^ and quoted as the most popular method within medical and dental schools in the United Kingdom.^24^, ^25^, ^26^

Longitudinal methods of assessment, however, bring their own challenges and deficiencies into the equation. They are usually expensive to set up, require extensive infrastructure and most importantly require a great deal of clinical supervisors’ buy‐in.^25^ This assessment method is heavily reliant on assessors’ willingness to be part of the assessment process and more importantly being able to make a sound judgment on the performance of their students. This on its own casts a shadow of doubt over validity and reliability of such an assessment method. Fraser's report confirmed that incompetent midwifery students ‘slip through the net’^27^ and initiate a ‘buzz’ of disbelief amongst many clinical professions.

Healthcare professionals such as medics,^28^, ^29^ nurses,^30^, ^31^, ^32^, ^33^, ^34^, ^35^, ^36^, ^37^, ^38^ social workers,^39^, ^40^ occupational therapists,^41^ and dentists^42^ have raised concerns that clinical modes of assessment can result in incompetent students graduating and being placed on healthcare professional registers.

The bachelor in dental surgery (BDS) program at the UK Dental School is designed based on a CBE curriculum with the current fluid concept of milestone assessments, where a student's clinical competence is evaluated throughout their undergraduate education.^3^ The emphasis placed on longitudinal assessment is regarded as a high‐stake mode of assessment, influencing the progression of students at each year, as their clinical progression as a whole is evaluated. The assessment data are captured using a commercial electronic portfolio system traded under the name of iDentity.^43^, ^44^, ^45^

iDentity allows tutors to grade the students on every single clinical encounter using one of the six possible grades: H, U, Sp, Sv, I and I+ (Table 1). All supervisors are briefed to make their judgment based on comparison with what they expect from a newly qualified dentist, therefore irrespective of the BDS year they are in. When processing the data at the Division level, grades of H and U are considered “fail” for the students in lower years whilst grades of H, U and Sp are considered “fail” for the students in the final year.

**TABLE 1:** iDentity marking descriptor

Scale	Title	Description
H	Harm	The student's performance, knowledge or action has resulted or could have resulted in harm to the patient, their relatives or members of staff.
U	Unsatisfactory	The student's performance, knowledge or action is below standards. However, this has not resulted in a compromise of the safety of the patients, their relatives or staff members.
Sp	Satisfactory— procedural intervention	The tutor (or other healthcare professional) has to intervene to produce a satisfactory outcome.
Sv	Satisfactory— verbal intervention	Satisfactory clinical outcome was achieved after verbal prompts from the tutor.
I	Independent	The student is capable of starting and finishing clinical tasks independently. The role of the tutor is purely supportive. No intervention from the tutor was needed.
I+	Excellent independence	The student's clinical work is beyond that expected in their foundation training year.

The iDentity system is heavily reliant on the judgment of tutors, many of whom are part‐time general dental practitioners (GDPs) who could be supervising the students for as little as half a day per week. Though tutors are invited to annual training on iDentity, there is little evaluation on the supervisors’ process of grading the students. Therefore, the aim of this research was to evaluate the failure to fail the students during clinical activity.

The objectives were:

- To understand tutors’ confidence in using the iDentity grading system.
- To understand if tutors are allowing failing the students to pass.
- To explore barriers why tutors may fail to fail poorly performing students.

## 2 METHOD

The study was completed at the UK Dental School. A short anonymised online survey was designed utilising themes in previously recorded literature of clinical assessment and distributed to all clinical supervisors in restorative dentistry. (*n* = 51) in June 2019. Ethical approval was not required following Research Ethics Committee review.

Demographics and teaching experience were ascertained, along with key questions on the utilisation of online assessment software iDentity, and the assessments process for tutor was explored. The options were populated from the most common features published in the literature, and free text was also utilised to discuss barriers they had experienced when grading the students.

### 2.1 Analysis of the data

The data collection was anonymised and recorded on Microsoft Excel 2010. Data analysis of the free‐text sections was completed using thematic analysis.^46^

## 3 RESULTS

**FIGURE 2:**
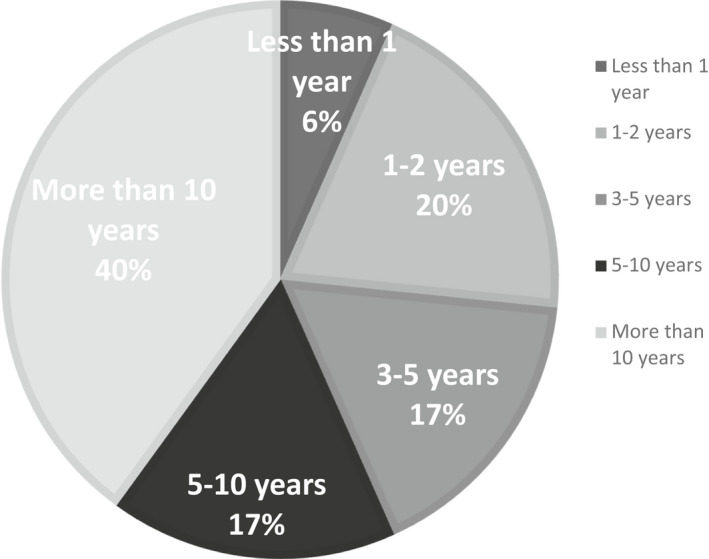
Experience of clinical supervisors teaching on RGFC

**FIGURE 3:**
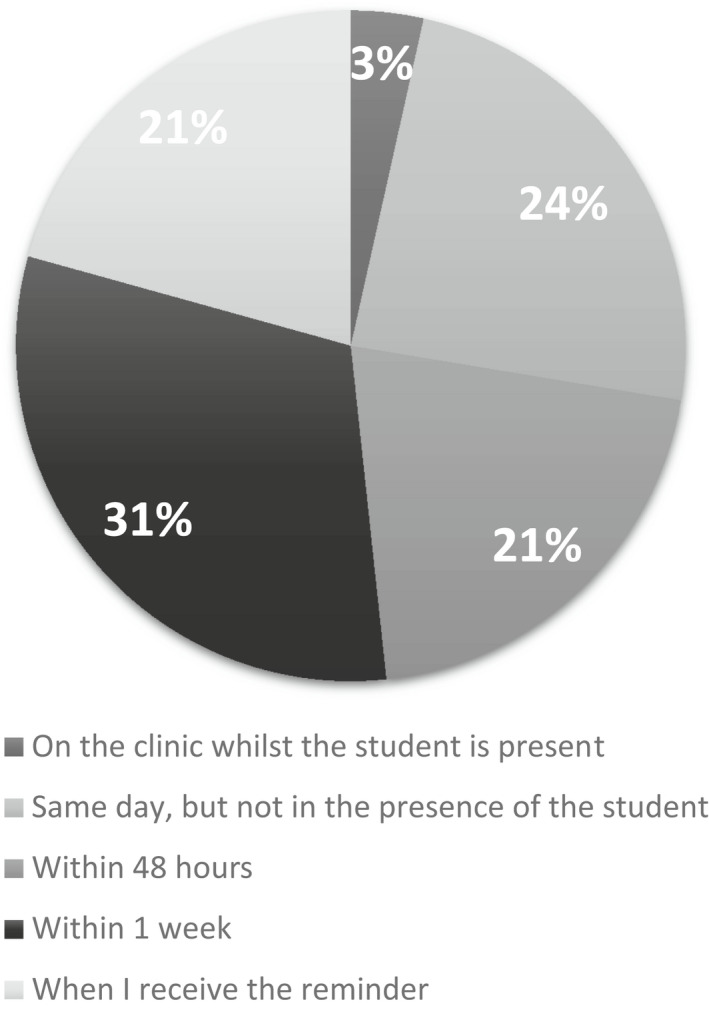
Time lag between clinical encounter and completion of grading

A total of 51 restorative staff were invited with a response rate of 59% (*n* = 30). One blank response was excluded. The staff who responded provided a representative sample of clinical supervision across all undergraduate years, with experience in clinical supervision ranging from >1 year with 40% of respondents recording over 10 years of clinical teaching experience (Figure 2). Less than half of the supervisors would complete the iDentity grading within 48 h of the clinical encounter. Just over 20% admitted that they complete the grading once they receive the automated email reminder, which is produced after 7 days (Figure 3). The number of clinical sessions (half a day) each tutor supervised ranged from 1–6 per week (2.3) average.

Whilst 87% of the tutors were confident with the grading system, the remaining lacked confidence in grading via iDentity. Thirty‐seven percent of the clinical supervisors admitted that they have allowed the failing students to pass on at least one occasion. Of the different possible reasons (tutors were able to select multiple answers), “giving the student the benefit of the doubt” and “considering the poor performance as a one‐off incident” were the most likely reasons with 63% and 59% respectively. This was followed by “good knowledge but inability to apply the knowledge to practice” (50%), “feeling uncomfortable to fail students” (33%), “lack of training on iDentity grade boundaries” (33%), “being afraid of negative consequences” (30%) and “aim to maintain good relationship with the student” (30%).

### 3.1 Barriers to failing students

A thematic analysis was undertaken by the free‐text comments, from which six themes emerged.

### 3.2 Maintaining good relationship

A key theme noted was the importance of having a good relationship with the students and the impact of providing the students with a grade of H or U. Several tutors’ responses used emotive language such as “hurt” or “worry” toward the student as a consequence when providing low grades on iDentity feedback:

*“Failing them with a bad mark after they have shown the effort to do things right will put them down instead to continue encourage to improve to do better.” (tutor 1–2 years’ experience)*.

*“I* *think sometimes we worry about knocking the student's confidence too severely.” (tutor 3–5 years’ experience)*.

*“Some students have a theoretical knowledge and need more practice to achieve satisfactory results.” (tutor 10+ years’ experience)*.

These responses highlight that some tutors view the grading as an emotional assessment, by not wanting to hurt the student's feelings. This results in prioritising avoidance of the potential negative impact on a student's confidence, if a poor grade was recorded, rather than providing an objective mark of the students’ clinical performance. Some tutors did not want to expose the students to the recorded consequence of failure, viewing verbal feedback sufficient in order to improve the student's clinical skill set and resolve the situation:

*“I have found it difficult on occasion, to give the students a lower grade because they may have prepared for the session and know what to do but have struggled on the clinic to perform the task. I don't want the student to feel upset that they have been marked down.” (tutor 1–2 years’ experience)*.

*“…They* *will instead receive a negative verbal feedback and so far, generally do not repeat the errors and things go better.” (tutor 5–10 years’ experience)*.

The avoidance of confrontation with the students was also highlighted as a barrier in providing the range of grades in the iDentity system. These views can illuminate the decision behind the grading and the avoidance of confrontation which staff may feel uncomfortable about, in turn protecting themselves, their relationship with the student and providing a positive grade as an ‘easier’ option. The students may be manipulative through questioning their grade, *becoming upset or confrontational*. *As a result*, *staff may be intimidated*:

*“…One* *student got very upset and wanted me to change it….” (tutor 1–2 years’ experience)*.

*“…It's* *easier to pass a student then than give negative feedback…” (tutor 3–5 years’ experience)*.

*“…[my] decision for the grade questioned….” (tutor 10 years’ experience)*.

The challenges of providing fail grades to students were also raised. Where instructions had not been followed or there was poor professional conduct, staff avoided low grades to prevent confrontation.

*“Work was of a good quality but professionally it was a fail, but I didn't want any repercussions and felt easier to grade as a pass.” (tutor 3–5 years’ experience)*.

*“Student not listening to me then continuing on with the root filing because they needed to finish it that session to get the grade. Student then got another tutor to help her and completed the work running into lunch time at 1pm.” (tutor 10+ years’ experience)*.

### 3.3 Limited supervision

Tutors raised concerns regarding their ability to appraise students’ knowledge from the single clinical session each week, whilst others commented on the clinical pressures resulting in difficulty to assess the student's competence and therefore provide an appropriate grade:

*“Only* *looking after students one day a week can be difficult to assess the students’ knowledge and then grade appropriately….” (tutor 3–5 years’ experience)*.

*“Being* *heavily booked on the clinic means that students can be left unsupervised….” (tutor 10+ years’ experience)*.

### 3.4 Time delay of grading

The completion of iDentity was a noted theme in the feedback, with a delay students’ part to upload their own responses onto iDentity after the procedure and tutors not having sufficient time to complete student feedback face to face:

***“*** *The time delay is too great for iDentity. I feel the assessment should take place on the clinic at the time of the procedure; however, the students often have not completed their score when I am on clinic and my response is done the following day. I feel feedback should be completed face to face with the student so the responses can be explained and discussed.” (tutor 10+ years’ experience)*.

*“I* *think that it would be better to feedback on the day and to create time to do so….” (tutor 5–10 years’ experience)*.

*“Insufficient* *time on clinic and students should have iPads signed in so you can feedback easily and immediately” (tutor 10+ years’ experience)*.

The perceived value of the iDentity process was also a theme noted by the staff, with limited understanding in the CBE process and its role in the students’ clinical progression. Another concern was noted that the tutor's views were dismissed by the academic staff:

*“…don't* *know to what extent the grades are looked at so unsure if further action is taken by the school….” (tutor 1–2 years’ experience)*.

*“…Tutors’* *concerns ultimately dismissed.“ (tutor 3–5 years’ experience)*.

It was also noted by tutors that the role of iDentity was only to record negatively if harm had been caused, even if the student had demonstrated inadequate clinical knowledge, rather than understanding the whole range of grades.

*“The* *most likely problem is that the student has poor knowledge and/or is ill prepared for the clinical session ‐ but no harm has been done.” (tutor 1–2 years’ experience)*.

*“I find it far more useful to discuss with the student on clinical any problems that may have occurred” (tutor >1 years’ experience)*.

### 3.5 A one‐off event

The students were given the benefit of the doubt on the procedure, as a one‐off event rather than recording the grade of the viewed procedure:

*“…when student started cleaning and shaping canals without WL xray. After discussing the situation with student it was clear it was a one off and she did understand the procedure. When a student was too concerned about finishing treatment quickly so can get on with final patient and expressed this in front of patient. Spoke to her after patient had left about what is not appropriate to say in front of patients.” (tutor 10 years’ experience)*.

*“I think there have been occasions when students have left an open contact in an MO, say, which is not a satisfactory conclusion but I’m not convinced it deserves an unsatisfactory grade. Similarly, with a slight ledge on a composite. If I had time to give more procedural help it would have been remedied. I would give a U if I thought the student was careless or failed to listen to instruction. I think I would give more Us to 4th or 5th years if required.” (tutor 10 years’ experience)*.

### 3.6 The first attempt

There were examples where suboptimal clinical performance was described when the students were completing the procedure for the first time. In these cases, the tutors showed empathy, as it was their first attempt, rather than providing an independent assessment of their clinical practice:

*“First restoration on a real patient. The only practice on the work was done in [clinical] skills. They know what to do but the scenario is different with more challenges. Generally, they are very scared and need a small push from tutor to exit from their comfort zone. They did not manage time correctly: took too long to apply Rubber dam and do LA leaving a small amount of time to complete the filling. They rush, as running out of time and made mistakes on the procedure that I had to correct in order to send the patient away safely. The overall grade would have been U but giving it to them on iDentity would have put them down. They definitively learned a lot from the experience in fact the next time think it went much much better.” (tutor 3–5 years’ experience)*.

*“A student completely over‐prepared a tooth for a 3/4 crown. The prep demonstrated good dexterity but lack of understanding and insight regarding the aim of the restoration. It was his first attempt, and I attributed the poor design to his inexperience.” (tutor >1 years’ experience)*.

### 3.7 Raising concerns

Ninety‐three percent felt comfortable raising concerns about a student, with two staff members feeling they were not comfortable. A free‐text section allowed the tutors to explore what pathways they would utilise in raising concerns regarding the failing students. The majority of responses noted they would liaise with the year lead regarding concerns about a student. Others noted they would explore concerns with another tutor before escalating. Two members of staff noted they would discuss the issue with the student initially, providing a warning verbally of potential provision of a “red card”, which is then raised with year lead. The final option discussed was to speak to the undergraduate director of the concerns regarding a student.

## 4 DISCUSSION

The emotional link between staff and students appears to dominate the feedback process, which is a common theme in clinical education literature.^47^ In turn, the students were potentially shielded from a true assessment of their clinical performance and limiting their development to independent clinical practice. By not wanting to expose the student to the consequence of failure, it could be argued as a conflict to the duty to protect the public. In examples where clinical care did not cause harm, but was not to a satisfactory standard, for example “over prepared crown”, “ledges” or “poor margins”. In these cases, is there a duty of candor to the patient to explain that the students’ performance was not satisfactory and to discuss the implications of this? Shielding the students from the consequence of failure and avoiding confrontation with the patient and student fail to develop on core skills required as a dental clinician and maintain good practice in line with the General Dental Council Standards. The systematic review by Yepes‐Rios (2016) highlighted that there is often parental approach by tutors when providing feedback to protect the students.^47^ Another barrier to clinical development is the tutors’ own person views on how they would be perceived by others for failing a student.^39^, ^41^, ^47^

A CBE program is heavily dependent on the tutors to perform an objective assessment of the student's performance. If the foundation of this assessment has a bias toward protection of the student and their perceived relationship, there is a risk that the students may slip through the net and graduate as students with significant deficiencies in their skill set that they were unaware of. To fail a student also required self believe and confidence in their own clinical judgment. Fear of legal action has been reported by dental, medical and nursing specialities during clinical education as a barrier to failing a student.^28^, ^41^, ^43^

The environmental constraints of the busy clinic and the ability to provide tailored feedback have been raised as a barrier using electronic assessments.^45^ The role of feedback as an appraisal and informational support was often overlooked due to time constraints, and completed days after the procedure had been undertaken, limiting the staff and student's reflection of their performance.

Only a small proportion of tutors provide students with immediate feedback on their clinical work. There are potential problems with this behavior, most importantly loss of valuable face‐to‐face feedback and also the potential for memory attrition. Therefore, the grade awarded may not be a true reflection of the student's performance. In improving the staff and student compliance in completing iDentity, the students must complete iDentity on clinic, with the gold standard of the tutors providing immediate feedback and recording on iDentity the same day.^45^ Where feedback is delayed, reducing the email reminder to daily rather than after one week may improve staff compliance. The delayed interaction with the software poses the question is the current evaluator tool a barrier to failing students? As noted by Bush et al (2013), where tools lack objectivity and explicit evaluation can reduce engagement for staff and thus value of tool.^42^ Tutors presented with a range of clinical experience in dental education, with several over 10 years of experience, demonstrate a bias to their own previous teaching styles and impact on engagement with software due to time contraints.^45^, ^47^

The utilisation of general dentists as tutors provides them with a range of clinical supervisors with experience outside the hospital setting. However, with some tutors only supervising half a session per week, interaction on an institutional training and the study highlighted further training opportunities that have been addressed. The need for continued assessment training is a current theme within clinical education to ensure the standards are maintained.^28^, ^43^

### 4.1 Limitations of the study

The authors acknowledge the limitations of the results as a single‐site evaluation, and further multicentred research would allow a more comprehensive overview on the current assessment process in dental education.

## 5 CONCLUSION

Continuous assessment of performance is a paramount pillar of competency‐based education and is utilised in undergraduate and postgraduate training within dental education. The study demonstrated a training needed in the utilisation of iDentity and understanding of its integral role in the assessment process and individual evaluator development. The utilisation of standardised software enables all students to be assessed against the same core standards providing high volume of data to evaluate student's progression across a range of clinical disciplines as a clinical logbook of competence. However, in order for the software to be utilised to its full potential, staff must be confident to fail and fairly grade the students on their clinical activity.

The desire for some staff to shield the students from failure often comes from a foundation of good intentions, trying to protect or motivate the student. However, if each tutor provides the student the benefit of the doubt, the student in turn is unable to assess their own weakness and believe themselves to be competent, and potentially overconfident.^28^, ^29^

Without this written evidence, the students may *“slip through the net”* with no supporting evidence of concern available at yearly student progression meetings. The study highlights the continued barriers within healthcare education, and the need continued the discussion for formalised training nationally in dental education. Due to this phenomenon, the students with significant incompetence (clinically or professionally) may become incompetent tutors when graduating. The need for honest and accurate assessment has a significant benefit to the student and staff, enabling targeted development to motivate the students and improve the quality of care provided to the patients.

## CONFLICT OF INTEREST

The authors declare that they have no conflict of interest.

## AUTHOR CONTRIBUTIONS

C Dixon and R Roudsari have both made substantial contributions to conception and design, or acquisition of data, or analysis and interpretation of data; and have been involved in drafting the manuscript or revising it critically for important intellectual content; and have given the final approval of the version to be published. Each author should have participated sufficiently in the work to take public responsibility for appropriate portions of the content and agreed to be accountable for all aspects of the work in ensuring that questions related to the accuracy or integrity of any part of the work are appropriately investigated and resolved.

## Data Availability

The data that support the findings of this study are available from the corresponding author upon reasonable request.

## References

[1] ADC. Professional competencies of the newly qualified dentist. Available at: http://www.ADC.org.au/documents/Professional%20Competencies%20of%20the%20Newly%20Qualified%20Dentist%20‐%20February%202016.pdf Pubished 2016 Accessed: 10 Jun 2019

[2] ADEA ‘Adea competencies for the new general dentist’. J Dent Educ. 2011;75(7):932‐935. 10.1002/j.0022-0337.2017.81.7.tb06299.x 31989605

[3] Frank JR , Snell L , Sherbino J . CanMEDS 2015 Physician Competency Framework. Royal College of Physicians and Surgeons of Canada; 2015.

[4] Combes JR , Arespacochaga E . Physician competencies for a 21st century health care system. J Grad Med Educ. 2012;4(3):401‐405. 23997896 10.4300/JGME-04-03-33 PMC3444207

[5] Cowpe J , Plasschaert A , Harzer W , Vinkka‐Puhakka H , Walmsley AD . Profile and competences for the graduating European dentist ‐ update 2009. Eur J Dent Educ. 2010;14(4):193‐202. 20946246 10.1111/j.1600-0579.2009.00609.x

[6] GDC. Standards for Education. Available at: https://www.GDC.‐uk.org/api/files/Standards%20for%20Education%20(v2%20revised%202015).pdf Published 2015; Accessed: 21 May 2020

[7] GMC Outcomes for graduates Available at. http://www.gmc‐uk.org/education/undergraduate/undergrad_outcomes.asp Published 2015, Updated 2016, Accessed: 21 May 2020

[8] Knowledge NDEB. Skills and Abilities (KSA) for the beginning general dentist. Available at: http://www.cdcr.ca.gov/career_opportunities/hr/ops/exams/analysis/pdfs/dentist/ksa.pdf Published 2014; Accessed: 2 Jun 2020

[9] Scottish Deans’ Medical Education Group The Scottish doctor: Learning Outcomes for the medical undergraduate in Scotland: A foundation for competent and reflective practitioners. Available at: http://www.scottishdoctor.org/resources/scotdoc3.pdf Published 2008, Accessed: 10 Jun 2019 10.1080/01421590220120713 12098432

[10] Aggarwal R , Darzi A . Technical‐skills training in the 21st century. N Engl J Med. 2006;355(25):2695‐2696. 17182997 10.1056/NEJMe068179

[11] Debas HT , Bass BL , Brennan MF . et al. American Surgical Association Blue Ribbon Committee Report on Surgical Education: 2004. Ann Surg. 2005;241(1):1–8. 15621984 10.1097/01.sla.0000150066.83563.52 PMC1356839

[12] Frank JR , Mungroo R , Ahmad Y , Wang M , De Rossi S , Horsley T . Toward a definition of competency‐based education in medicine: a systematic review of published definitions. Med Teach. 2010;32(8):631‐637. 20662573 10.3109/0142159X.2010.500898

[13] Miller GE . The assessment of clinical skills/competence/performance. Acad Med. 1990;65(9):S63‐S67. 2400509 10.1097/00001888-199009000-00045

[14] Epstein RM . Assessment in medical education. N Engl J Med. 2007;356(4):387‐396. 17251535 10.1056/NEJMra054784

[15] Epstein RM , Hundert EM . Defining and assessing professional competence. JAMA. 2002;287(2):226‐235. 11779266 10.1001/jama.287.2.226

[16] Hager P , Gonczi A , Athanasou J . General Issues about Assessment of Competence. Assess & Evaluation Higher Edu. 2006;19(1):3‐16.

[17] Hodges B , Regehr G , McNaughton N , Tiberius R , Hanson M . OSCE checklists do not capture increasing levels of expertise. Acad Med. 1999;74(10):1129‐1134. 10536636 10.1097/00001888-199910000-00017

[18] Kane MT . The Assessment of Professional Competence. Eval Health Prof. 1992;15(2):163‐182. 10119160 10.1177/016327879201500203

[19] LaDuca A , Taylor DD , Hill IK . The design of a new physician licensure examination. Eval Health Prof. 1984;7(2):115‐140. 10267247 10.1177/016327878400700201

[20] McMullan M , Endacott R , Gray MA , et al. Portfolios and assessment of competence: a review of the literature. J Adv Nurs. 2003;41(3):283‐294. 12581116 10.1046/j.1365-2648.2003.02528.x

[21] van der Vleuten CPM . The assessment of professional competence: developments, research and practical implications. Adv Health Sci Educ. 1996;1(1):41‐67. 10.1007/BF00596229 24178994

[22] Albino JE , Young SK , Neumann LM , et al. Assessing dental students’ competence: best practice recommendations in the performance assessment literature and investigation of current practices in predoctoral dental education. J Dent Educ. 2008;72(12):1405‐1435. 19056620

[23] Kramer GA , Albino JE , Andrieu SC , et al. Dental student assessment toolbox. J Dent Educ. 2009;73(1):12‐35. 19126764

[24] Brown HJ , Miles PV , Perelman RH , Stockman JA . A continuum of competency assessment: the potential for reciprocal use of the Accreditation Council for Graduate Medical Education toolbox and the components of the American Board of Pediatrics Maintenance‐of‐Certification Program. Pediatrics. 2009;123(1):S56‐S58. 19088247 10.1542/peds.2008-1578M

[25] Prescott LE , Norcini JJ , McKinlay P , Rennie JS . Facing the challenges of competency‐based assessment of postgraduate dental training: Longitudinal Evaluation of Performance (LEP). Med Educ. 2002;36(1):92‐97. 11849528 10.1046/j.1365-2923.2002.01099.x

[26] Prescott‐Clements L , van der Vleuten CPM , Schuwirth LWT , Hurst Y , Rennie JS . Evidence for validity within workplace assessment: the Longitudinal Evaluation of Performance (LEP). Med Educ. 2008;42(5):488‐495. 18298449 10.1111/j.1365-2923.2007.02965.x

[27] Fraser D , Murphy R , Worth‐Butler M . (1998). Preparing Effective Midwives: an outcome evaluation of the effectiveness of pre‐registration midwifery programmes of education: English National Board for Nursing, Midwifery and Health Visiting.

[28] Cleland JA , Knight LV , Rees CE , Tracey S , Bond CM . Is it me or is it them? Factors that influence the passing of underperforming students. Med Educ. 2008;42(8):800‐809. 18715477 10.1111/j.1365-2923.2008.03113.x

[29] Monrouxe LV , Rees CE , Lewis NJ , Cleland JA . Medical educators’ social acts of explaining passing underperformance in students: a qualitative study. Adv Health Sci Educ Theory Pract. 2011;16(2):239‐252. 21063770 10.1007/s10459-010-9259-y

[30] Black S , Curzio J , Terry L . Failing a student nurse: a new horizon of moral courage. Nurs Ethics. 2014;21(2):224‐238. 23989859 10.1177/0969733013495224

[31] Docherty A , Dieckmann N . Is there evidence of failing to fail in our schools of nursing? Nursing Edu Perspectives. 2015;36(4):226‐231. 10.5480/14-1485 26328290

[32] Heaslip V , Scammell JM . Failing underperforming students: the role of grading in practice assessment. Nurse Educ Pract. 2012;12(2):95‐100. 21907621 10.1016/j.nepr.2011.08.003

[33] Jervis A , Tilki M . Why are nurse mentors failing to student nurses who do not meet clinical performance standards?' British Journal of Nursing. 2011;20(9):582‐587. 21647023 10.12968/bjon.2011.20.9.582

[34] Killam LA , Montgomery P , Luhanga FL , Adamic P , Carter LM . Views on unsafe nursing students in clinical learning. Int J Nurs Educ Scholarsh. 2010;7(1):p. Article36. 10.2202/1548-923X.2026 21044036

[35] Lankshear A . Failure to fail: the teacher's dilemma. Nurs Stand. 1990;4(20):35‐37. 10.7748/ns.4.20.35.s33 2107445

[36] Luhanga F , Yonge OJ , Myrick F . "Failure to Assign Failing Grades": Issues with Grading the Unsafe Student. Int J Nursing Edu Scholarsh. 2008;5(1):1‐14. 10.2202/1548-923X.1366 18384275

[37] Scholes J , Albarran J . Failure to fail: facing the consequences of inaction. Nurs Crit Care. 2005;10(3):113‐115. 15918422 10.1111/j.1362-1017.2005.00118.x

[38] Shapton M . Failing to fail students in the caring professions. The Journal of Practice Teaching and Learning. 2012;7(2):39‐54.

[39] Finch J , Taylor I . Failure to fail? Practice educators’ emotional experiences of assessing failing social work students. Social Work Education. 2013;32(2):244‐258.

[40] Furness S , Gilligan P . Fit for purpose: issues from practice placements, practice teaching and the assessment of students’ practice. Social Work Education. 2004;23(4):465‐479.

[41] Ilott I , Murphy R . Feelings and failing in professional training: the assessor's dilemma. Asses Eval Higher Edu. 1997;22(3):307‐316.

[42] Bush HM , Schreiber RS , Oliver SJ . Failing to fail: clinicians’ experience of assessing underperforming dental students. Eur J Dent Educ. 2013;17(4):198‐207. 24127760 10.1111/eje.12036

[43] Ellis J , Teasdale D , Cotterill S , Thomason J . Is a generic UK e‐portfolio for dentistry desirable and achievable? Eur J Dent Educ. 2010;14(4):254‐256. doi:10.1111/j.1600-0579.2010.00630.x 20964091

[44] iDentity. iDentity: ePortfolio and assessment for Dentistry. Available at: http://www.eportfolios.ac.uk/dentistry Published 2017; (Accessed: 30 March 2019)

[45] Vernazza C , Durham J , Ellis J , et al. Introduction of an e‐portfolio in clinical dentistry: staff and student views. Eur J Dent Educ. 2011;15(1):36‐41. doi:10.1111/j.1600-0579.2010.00631.x 21226804

[46] Braun V , Clarke V . Using thematic analysis in psychology. Qual Res Psychol. 2006;3(2):77‐101. doi:10.1191/1478088706qp063oa

[47] Yepes‐Rios M , Dudek N , Duboyce R , Curtis J , Allard RJ , Varpio L . The failure to fail underperforming trainees in health professions education: A BEME systematic review: BEME Guide No. 42. Med Teach. 2016;38(11):1092–1099. 10.1080/0142159X.2016.1215414 27602533

